# Choledochal Cyst and Chronic Pancreatitis — Treated by Proximal Pancreatectomy

**DOI:** 10.1155/1991/38109

**Published:** 1991

**Authors:** R. P. Jalleh, R. C. N. Williamson

**Affiliations:** Department of Surgery Royal Postgraduate Medical School Hammersmith Hospital Du Cane Road London W12 0NN UK

## Abstract

A 32-year-old woman with a choledochal cyst (Todani type I) developed recurrent acute pancreatitis leading to calcific chronic pancreatitis. She had previously been treated with two cyst drainage procedures and subtotal cyst excision. This association between choledochal cyst and chronic pancreatitis has not been previously reported. Severe continuing symptoms led to pylorus-preserving proximal pancreatoduodenectomy, which was undertaken to prevent future carcinoma in the cyst remnant and progression of the chronic pancreatitis


HPB Surgery, 1991, Vol. 4, pp. 245–250
Reprints available directly from the publisher
Photocopying permitted by license only

© 1991 Harwood Academic Publishers GmbH
Printed in the United Kingdom

### CASE REPORT

### CHOLEDOCHAL CYST AND CHRONIC PANCREATITIS — TREATED BY PROXIMAL PANCREATECTOMY

R.P. JALLEH and R.C.N. WILLIAMSON
*Department of Surgery, Royal Postgraduate Medical School, Hammersmith Hospital, Du Cane Road, London, UK*

*(Received 10 December 1990)*

A 32-year-old woman with a choledochal cyst (Todani type I) developed recurrent acute pancreatitis leading to calcific chronic pancreatitis. She had previously been treated with two cyst drainage procedures and subtotal cyst excision. This association between choledochal cyst and chronic pancreatitis has not been previously reported. Severe continuing symptoms led to pylorus-preserving proximal pancreatoduodenectomy, which was undertaken to prevent future carcinoma in the cyst remnant and progression of the chronic pancreatitis.

KEY WORDS: Choledochal cyst, chronic pancreatitis, pylorus preserving proximal pancreatoduodenectomy

### INTRODUCTION

The incidence of choledochal cyst in Western countries is estimated at one in every 100,000–150,000 live births[1]. There is a female preponderence of nearly 4:1[2]. Complications of choledochal cyst in adult life include gallstones, cholangitis, cholangiocarcinoma and pancreatitis[3,4]. In Yamaguchi's collected series of 1433 patients from Japan, there were six cases of pancreatitis of unspecified type[5]. We have recently reported two patients with recurrent acute pancreatitis[4], and this association is well recognised though uncommon[2,6,7]. Although chronic calcific pancreatitis can be the end stage of recurrent acute attacks[8], it does not appear to have been reported as a complication of choledochal cyst. We now describe such an association in a young woman.

### CASE REPORT

A 32-year-old woman was referred to this hospital after four previous operations for choledochal cyst. At the age of 3 months and again at 12 years, she had undergone a cyst drainage operation. Thereafter she was well until the age of 30

Address correspondence to: Professor R.C.N. Williamson, Department of Surgery, Royal Postgraduate Medical School, Hammersmith Hospital, Du Cane Road, London W12 0NN, UK.

years, when repeated episodes of cholangitis led to subtotal excision of the cyst with Roux-en-Y hepaticojejunostomy. Soon afterwards attacks of acute pancreatitis commenced, each time with marked elevation of serum amylase. An endoscopic retrograde cholangiopancreatogram (ERCP) showed a 5mm calculus in the pancreatic duct in the head of pancreas, and further operation was undertaken. Despite a wide transduodenal sphincteroplasty, the orifice of the pancreatic duct could not be reached nor could the stone be extracted. By the time of referral to the Hammersmith Hospital, she had had at least four further attacks of documented acute pancreatitis plus numerous minor episodes. Alcohol intake had never exceeded 3-4 units per week nor had gallstones ever been found.

Normal investigations included liver function tests, serum amylase, glucose tolerance test and pancreolauryl ratio for exocrine pancreatic function. Computed tomography showed dilatation of the residual intrapancreatic bile duct and a focus of calcification in the pancreatic head. Repeat ERCP confirmed a sizeable remnant of choledochal cyst plus two calculi partly obstructing the pancreatic duct (Figure 1a,b).

Resection of the pancreatic head seemed the best approach for this complex problem. A 3 cm remnant of the choledochal cyst was dissected free from the Roux loop leading to the biliary anastomosis and from the hepatic artery and portal vein. Following pylorus-preserving proximal pancreatoduodenectomy[9], a retrograde pancreatogram from the neck showed dilatation of the distal pancreatic duct, but the body and tail of pancreas appeared much less severely diseased than the head. The previous hepaticojejunostomy was not disturbed, so reconstruction involved end-to-end pancreatojejunostomy and end-to-side duodenojejunostomy (Figure 2a,b). Recovery was uneventful and postoperative glucose tolerance test was unchanged. Since the pancreolauryl ratio had fallen from 35% to 7% (normal >30%), pancreatic enzyme therapy was started postoperatively. Nine months later she is well and free of pain.

The resected specimen showed an anomalous pancreatobiliary ductal junction (PBDJ). The common channel measured 20 mm in length. It received an inflamed, dilated main pancreatic duct containing the two stones and, via a pin-point orifice, the remaining portion of the choledochal cyst. The cyst showed no evidence of malignancy. There was clear-cut chronic pancreatitis in the head of pancreas with focal areas of active inflammation.

### DISCUSSION

The association between cystic dilatation of the biliary tree and an anomalous PBDJ has been clearly established by Japanese workers[10-12]. The abnormal common channel, which is >15 mm long instead of the normal <5 mm, probably allows reciprocal reflux of bile and pancreatic juices[12]. Amylase can be shown in the bile and may explain the increased incidence of cholangiocarcinoma and gallbladder carcinoma[13,14]. As a corollary, entry of bile into the pancreatic duct might lead to recurrent acute pancreatitis with the eventual development of chronic inflammation, fibrosis and calcification of the pancreas[8]. Surprisingly, we can find no previous report of a patient with choledochal cyst and calcific chronic pancreatitis.

Excision of a choledochal cyst is nearly always preferable to simple drainage, which runs the risk of recurrent cholangitis and carcinoma[3,15]. In our patient, the

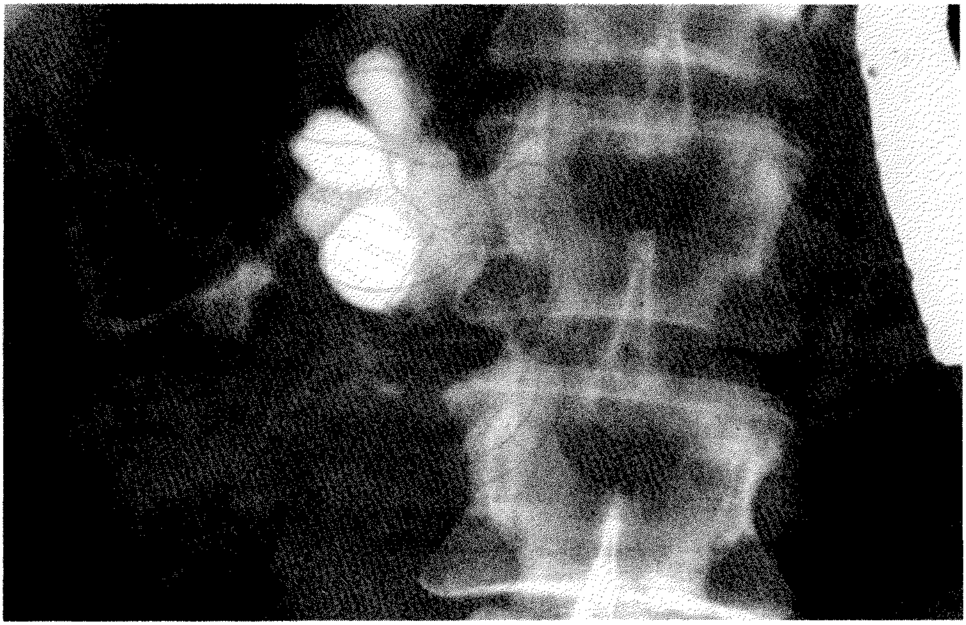

**Figure 1a** ERCP showing lobulated remnant choledochal cyst.

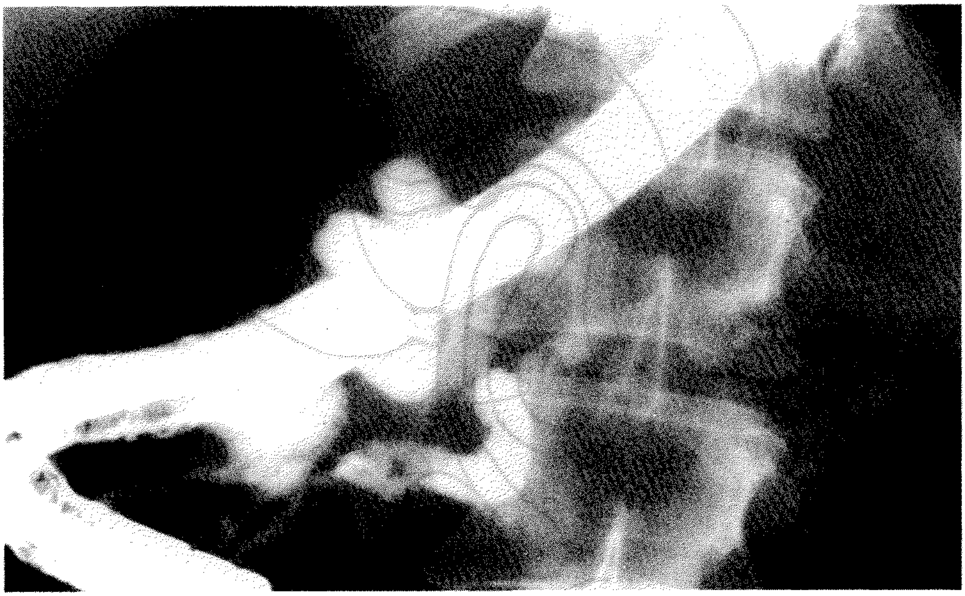

**Figure 1b** Dilated pancreatic duct with calculus (arrow).

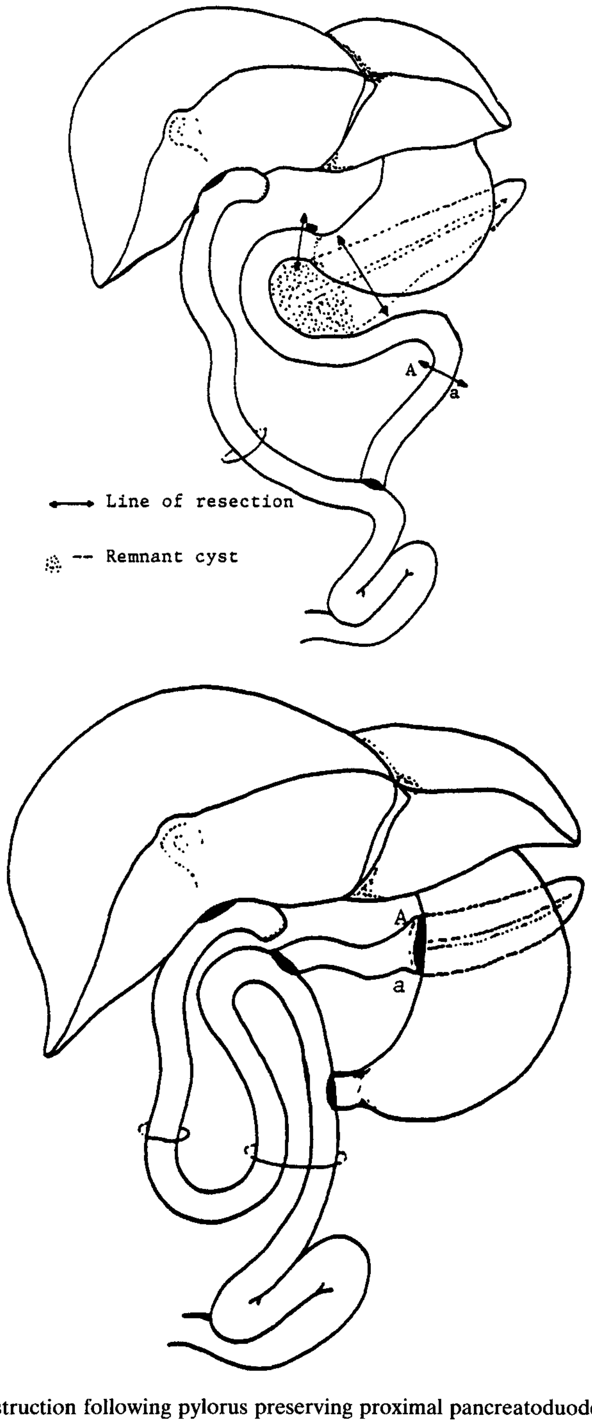

**Figure 2a,b** Reconstruction following pylorus preserving proximal pancreatoduodenectomy.

cyst excision performed 17 years after the second drainage procedure succeeded in preventing further cholangitis but seemed to precipitate the development of acute-on-chronic pancreatitis. Perhaps the intrapancreatic cyst remnant caused stasis of pancreatic juice and led to calculus formation in the duct. The extent of chronic pancreatitis is manifested by the fact that a limited pancreatectomy (40–50 per cent) has led to near-total exocrine insufficiency.

In Todani's series of 73 patients with excision of choledochal cyst, seven patients needed reoperation for bleeding, cholangitis or anastomotic leakage[15]. Two patients developed acute pancreatitis but neither required reoperation. Thus, pancreatitis represented a unique indication for reoperation after cystectomy in our patient. By removing the cyst remnant and the obstructing pancreatic calculi, conservative proximal pancreatectomy should prevent future carcinoma and progression of chronic pancreatitis.

### *References*

1. Howard, E.R. (1985) Choledochal cysts. In: Schwartz, S.I., Ellis, H. (eds) *Maingot's Abdominal Operation*. 8th Edn. Norwalk, Connecticut: Appleton-Century-Crofts
2. Tan, K.C., Howard, E.R. (1988) Choledochal cyst: a 14 year surgical experience with 36 patients. *British Journal of Surgery*, **75**, 892–895
3. Traverso, L.W. (1989) Biliary surgery: congenital and trauma. *Current Opinion in Gastroenterology*, **5**, 658–661
4. Hopkins, N.F.G., Benjamin, I.S., Thompson, M.H. and Williamson, R.C.N. (1990) Complications of choledochal cysts in adulthood. *Annals of the Royal College of Surgeons of England*, **72**, 229–235
5. Yamaguchi, M. (1980) Congenital choledochal cysts: Analysis of 1433 patients in the Japanese literature. *American Journal of Surgery*, **140**, 653–657
6. Altman, M.S., Halls, J.M., Douglas, A.P. and Renner, I.G. (1978) Choledochal cyst presenting as acute pancreatitis: evaluation with ERCP. *American Journal of Gastroenterology*, **70**, 514–519
7. Cuschieri, A. and Davies, R.S. (1969) Acute pancreatitis complicating a choledochal cyst. *British Medical Journal*, **iii**, 109
8. Williamson, R.C.N. and Cooper, M.J. (1987) Resection in chronic pancreatitis. *British Journal of Surgery*, **74**, 807–812
9. Cooper, M.J. and Williamson, R.C.N. (1985) Conservative pancreatectomy. *British Journal of Surgery*, **72**, 801–803
10. Ono, J., Sakoda, K. and Akita, H. (1982) Surgical aspects of cystic dilatation of the bile ducts. An anomalous junction of the pancreatobiliary tract in adults. *Annals of Surgery*, **195**, 203--208
11. Todani, T., Watanabe, Y., Fujii, T. and Uemera, S. (1984) Anomalous arrangement of the pancreatobiliary ductal system in patients with a choledochal cyst. *American Journal of Surgery*, **147**, 672–676
12. Oguchi, Y., Okada, A., Nakamura, T. *et al.* (1988) Histopathologic studies of congenital dilatation of the bile duct as related to an anomalous junction of the pancreatobiliary ductal system: clinical and experimental studies. *Surgery*, **103**, 168–173
13. Nagata, E., Sakai, K., Kinoshita, H. and Hironoshi, K. (1986) Choledochal cyst: complications of anomalous connection between the choledochal and pancreatic duct and carcinoma of the biliary tree. *World Journal of Surgery*, **10**, 102–110
14. Yamauchi, S., Kage, A., Matsumato, S., Tanaka, M. and Nakayama, F. (1987) Anomalous junction of the pancreatobiliary duct without congenital choledochal cyst: a possible risk factor for gallbladder cancer. *American Journal of Gastroenterology*, **82**, 20–24
15. Todani, T., Watanabe, Y., Toki, A., Urushihara, N. and Sato, Y. (1988) Reoperation for congenital choledochal cyst. *Annals of Surgery*, **207**, 142–147

*(Accepted by S.Bengmark 13 March 1991)*

### INVITED COMMENTARY

The association between attacks of acute pancreatitis and the presence of a choledochal cyst is now well recognized and is usually related to the presence of a 'common' pancreatico-biliary channel at the ampulla. Babbitt (1969)[1] first described common channel between the lower end of the bile duct and the pancreatic duct in 19 cases of choledochal cyst and he suggested that pancreatic reflux might damage the common bile duct and cause the dilatation. These anomalous junctions have now been described in many reports and in a personal series of 39 cases no less than 29 had long common channels and high levels of amylase within the bile.

The pancreatitis in these patients should be differentiated from so-called fictitious pancreatitis. Free reflux of pancreatic juice can occur through any common pancreatic biliary channel and amylase may be absorbed through the choledochal cyst wall which usually has an incomplete epithelial lining. Hyper-amylasaemia may therefore be detected without the inflammation of the gland. We have also seen recurrent pancreatitis in 3 out of 4 cases treated with cyst enterostomy, rather than radical cyst excision, and all of these had a common pancreatico-biliary channel.

Diversion of bile by cyst excision and Roux loop reconstruction usually prevents any further episodes of pancreatitis. The present case report is therefore unusual. Firstly, because bile drainage had been diverted previously and secondly because the patient developed a chronic form of pancreatitis. There was however partial obstruction by calculi in the pancreatic duct and a retained portion of choledochal cyst behind the duodenum which may have harboured infection. Both of these factors have been implicated in the aetiology of pancreatitis (Schmidt and Creuzfeldt, 1976).

### *References*

1. Babbitt, D.P. (1969) Congenital choledochal cyst: new etiological concept based on anomalous relationship of the common bile duct and pancreatic bulb. *Annals of Radiology*, **12**, 231–240
2. Schmidt, H., Creuzfeldt, W. (1976) Etiology and pathogenesis of pancreatitis. In: *Gastroenterology*, edited by H.L.Bockus, p.1007, Philadelphia: W.B.Saunders

Mr. Edward R. Howard
Department of Surgery
King's College Hospital
Denmark Hill
LONDON 8E5 9RS
UK

